# AI redefines mass spectrometry chemicals identification: retention time prediction in metabolomics and for a Human Exposome Project

**DOI:** 10.3389/fpubh.2025.1687056

**Published:** 2025-11-12

**Authors:** Fenna C. M. Sillé, Carsten Prasse, Thomas Luechtefeld, Thomas Hartung

**Affiliations:** 1 Center for Alternatives to Animal Testing (CAAT), Department of Environmental Health and Engineering, Bloomberg School of Public Health and Whiting School of Engineering, Johns Hopkins University, Baltimore, MD, United States; 2 Department of Environmental Health and Engineering, Bloomberg School of Public Health and Whiting School of Engineering, Johns Hopkins University, Baltimore, MD, United States; 3 Bloomberg School of Public Health, Risk Sciences and Public Policy Institute, Johns Hopkins University, Baltimore, MD, United States; 4 Insilica Inc., Rockville, MD, United States; 5 CAAT-Europe, University of Konstanz, Konstanz, Germany

**Keywords:** untargeted metabolomics, retention time prediction, quantitative structure–retention relationships, artificial intelligence, exposomics, environmental health, Human Exposome Project

## Abstract

The comprehensive identification of environmental and endogenous chemicals in human biospecimens is a critical bottleneck for realizing the Human Exposome Project. Untargeted metabolomics, particularly liquid chromatography–high-resolution mass spectrometry (LC–HRMS), offers unparalleled coverage of small molecules, but most detected features remain unidentified due to limited spectral libraries and structural ambiguity. Retention time (RT) prediction—based on quantitative structure–retention relationships (QSRR) and enhanced by artificial intelligence (AI)—is an underutilized orthogonal parameter that can substantially improve metabolite annotation confidence. This review synthesizes advances in machine learning–based RT prediction, probabilistic calibration, and cross-platform harmonization for liquid chromatography and gas chromatography, including deep learning, graph neural networks, and transfer learning approaches. We evaluate workflows integrating RT prediction with mass-based searches and network-based annotation tools, highlighting their potential to refine candidate ranking and reduce false positives in environmental exposure assessment. The use of endogenous compounds as internal calibrants is discussed as a practical strategy for improving RT transferability across laboratories. We further outline how RT-aware annotation supports non-targeted screening of emerging contaminants, transformation products, and exposure biomarkers, thereby enhancing the interpretability and reproducibility of exposomics data. By integrating RT prediction, QSRR modeling, and AI into untargeted metabolomics pipelines, researchers can move from qualitative detection toward quantitative, inference-driven mapping of environmental influences on human health, strengthening the scientific foundation for environmental health policy and preventive public health strategies.

## Introduction

Exposomics (1) and in consequence a possible Human Exposome project (2) depend critically on the identification of metabolites in human biofluids. Untargeted metabolomics of human blood yields vast numbers of features detected by liquid or gas chromatography coupled with high-resolution mass spectrometry (LC–HRMS and GC–HRMS). A persistent challenge is the identification of unknown metabolites from these features. Researchers typically rely on measuring the mass spectrometry (MS) mass-to-charge ratios (m/z) of molecular ions and fragmentation spectra (MS/MS) with accurate mass to search databases for candidate structures (3). However, many features remain unidentified because multiple compounds can share the same m/z or similar spectra (4, 5) and a main limitation is also that the MS and MS/MS databases are still very limited. An often underused piece of information is the chromatographic retention time (RT) at which a compound elutes. RT is strongly influenced by molecular structure and physicochemical properties, making it a valuable orthogonal feature for narrowing candidate lists (5). Integrating RT prediction, a type of quantitative structure–retention relationship (QSRR), with mass-based searches and machine learning (ML) offers a promising route to improve unknown identification. As Kaliszan (6) outlined in his seminal review, retention is governed by thermodynamically driven interactions between analytes and chromatographic phases. QSRR models translate these interactions into predictive equations using descriptors such as molar volume, polarizability, and charge distribution. The extrathermodynamic framework he proposed remains foundational for understanding retention modeling in metabolomics workflows. As Héberger (7) outlines in his comprehensive review of QSRR practices, robust model performance hinges on clear definition of applicability domain, independent test validation, and proper residual analysis. Overfitting, data leakage during feature selection, and misuse of correlation coefficients continue to plague the field. These concerns are especially salient when applying QSRR models to untargeted data across laboratories with varying chromatographic conditions. To democratize QSRR modeling, Naylor et al. (8) developed QSRR Automator, a Graphical User Interface (GUI) based tool that enables rapid construction of retention time models with performance comparable to expert-curated algorithms. It supports common ML methods such as Support Vector Regression (SVR), Random Forest (RF), and Multiple Linear Regression (MLR) and accommodates varying LC conditions, making it ideal for multi-lab exposomic studies with high throughput needs.

RT information, when used properly, can filter out false candidates and boost annotation accuracy. Historically, RT usage has been limited because experimental RT data are sparse in metabolomic databases and RTs vary widely between labs and chromatographic methods. Recent advances address these issues: large RT datasets (e.g., METLIN’s 80 k-compound Small Molecule RT dataset) have enabled accurate QSRR models, and new calibration techniques correct RT differences between instruments. Furthermore, modern artificial intelligence (AI) and deep learning methods can predict RT and related properties with high accuracy, and these predictions can be incorporated into metabolite identification workflows. In this report, we evaluate how RT prediction improves candidate ranking, discuss strategies to calibrate RT across different runs and chromatographic modalities, review relevant AI/ML approaches and tools, and outline best practices for applying this integrated approach to current and future metabolomics studies.

Retention time, indicative of the compound’s physicochemical interaction with the chromatographic stationary and mobile phases, serves as an orthogonal structural descriptor that can complement m/z of MS and MS/MS information. RT narrows the plausible candidate space and provides additional discrimination among structural isomers, even in the absence of fragmentation data. However, until recently, RT data was often ignored or used only heuristically due to variability across platforms and lack of predictive tools. Recent advances in AI-based QSRR have transformed this landscape. These ML and deep learning models—especially those using molecular fingerprints, graph neural networks, or transformer architectures—can now predict RT with impressive accuracy. Despite these advancements, the broader metabolomics community has been slow to adopt RT-aware workflows as standard practice. This is due in part to the fragmented nature of prediction tools, lack of calibration protocols, and insufficient cross-platform harmonization. In addition, retention time prediction models often perform optimally only under specific chromatographic conditions unless fine-tuned or recalibrated. For exposome research, where retrospective data integration across diverse platforms is essential, this variability becomes a critical limitation. Standardization of RT prediction models and incorporation into open-source tools and FAIR-compliant pipelines is needed.

The lack of high-confidence identification in untargeted metabolomics is more than a technical limitation—it is a rate-limiting step for the exposome field. Without precise structural information, linking exposures to molecular mechanisms or population health outcomes remains speculative. Moreover, annotation uncertainty hampers reproducibility across labs and reduces confidence in derived biomarkers. The inability to fully interpret the metabolome limits both hypothesis-driven toxicology and the discovery of unanticipated environmental contributors to disease.

To realize the Human Exposome Project, we must therefore invest in RT-informed annotation workflows as standard practice. This includes widespread adoption of QSRR models, routine use of internal calibrants in LC–MS and GC–MS workflows, and integration of RT prediction into automated annotation pipelines. As RT-aware identification matures, it will bridge the gap between feature detection and actionable insights—transforming untargeted metabolomics from an exploratory tool into a quantitative engine for environmental health science.

## Background exposomics and a possible Human Exposome Project

From Genome to Exposome: The completion of the Human Genome Project in 2003 marked a watershed moment in biomedical science, successfully mapping all human genes at a cost of $3 billion and generating an economic impact exceeding $965 billion by 2010.^1^ However, despite this monumental achievement, a critical gap remained in our understanding of disease causation: while estimates vary, analyses suggest that heritable genetic factors may account for only a small fraction of chronic disease risk (on the order of a few percent), whereas environmental exposures and other non-genetic factors could contribute the majority (on the order of tens of percent to over half) (9). For example, one analysis attributed ~70–90% of chronic disease risk to differences in environmental exposure (9), underscoring the need to map the ‘exposome’ alongside the genome.

This realization has catalyzed the emergence of exposomics—the comprehensive study of environmental exposures and their biological effects throughout the human lifespan. As Wild (10) first articulated, complementing the genome with an “exposome” represents “*the outstanding challenge of environmental exposure measurement in molecular epidemiology*.” The exposome encompasses all physical, chemical, biological, and psychosocial factors that individuals encounter from conception to death, excluding DNA sequence variation.

### The exposome concept and environmental health

The exposome framework provides a systematic approach to understanding how environmental factors contribute to disease development, making it particularly relevant for analyzing emerging contaminants and the environmental exposome (11). Unlike traditional toxicological approaches that focus on single compounds, exposomics embraces the complexity of real-world exposure scenarios where individuals encounter multiple chemicals simultaneously through various pathways.

Environmental exposures encompass the general external environment (pollution, climate, built environment), specific external factors (lifestyle, occupation, diet, infections), and the internal environment (metabolism, inflammation, oxidative stress, aging). Exposomics therefore aims to measure xenobiotics and endogenous metabolites, i.e., to identify exposure and its imprint on metabolism; noteworthy, gene expression to characterize the perturbation of biology and possibly indications of adverse reactions (hazard manifestations) also for a crucial tool to form an exposure hypothesis connecting to disease (1). This comprehensive framework is essential for understanding how emerging environmental contaminants—including pharmaceuticals, personal care products, industrial chemicals, and their transformation products—contribute to the growing burden of chronic disease.

### The need for advanced analytical approaches

Traditional environmental monitoring approaches that rely on targeted analysis of known compounds are insufficient for characterizing the full scope of environmental exposures. The chemical universe contains over 200 million registered substances, with approximately 80,000 requiring investigation for potential environmental and human health risks according to regulatory agencies. Most chemicals present in environmental and biological samples remain structurally unknown (≤1% have been characterized). This challenge necessitates advanced analytical platforms capable of non-targeted screening to detect previously unknown contaminants, suspect screening to identify compounds of emerging concern, and metabolomics approaches to understand biological effects of exposures. The advancements modern HRMS instruments allow us to tackle all of these aspects in a single analytical run. Another critical advancement is the integration of retention time prediction with quantitative structure–activity relationships (QSAR) and AI, enabling more confident identification of unknown environmental chemicals in biological samples.

### The HEP vision

Building on the success and lessons learned from the Human Genome Project, the scientific community has begun organizing around the concept of a Human Exposome Project (HEP). This ambitious initiative aims to comprehensively map environmental exposures and their health effects with the same rigor and scale that characterized genomic mapping efforts. We started a project to realize this vision (12, 58).

### Key components of the human exposome project

Three key technical components toward HEP were identified:

#### Global research infrastructure

The project leverages existing international networks including the European Exposome Infrastructure (EIRENE),^2^ backed by 17 EU governments with over €1 billion in projected investments, the International Human Exposome Network (IHEN),^3^ the Network for Exposomics in the United States (NEXUS),^4^ and emerging partnerships across Africa, Asia, and Latin America.

#### Technological integration

The HEP platform integrates high-throughput mass spectrometry for chemical analysis, wearable biosensors for real-time exposure monitoring, geospatial mapping for environmental context, artificial intelligence for pattern recognition and prediction, and multi-omics integration across genomics, transcriptomics, proteomics, and metabolomics.

#### AI-driven knowledge creation

AI serves as the “Apollo Guidance Computer” of the exposome moonshot (13), enabling faster data interpretation and hypothesis generation, cost reduction through automation and reduced animal testing, democratized access to analytical capabilities for low-resource settings, and real-time synthesis of complex, heterogeneous datasets.

#### The Washington declaration and global momentum

Two of the authors (FS, TH) were the hosts of the inaugural Human Exposome Moonshot Forum (Washington, D.C., May 2025),^5^ which brought together over 300 scientists, policymakers, ethicists, and civil society representatives from 50+ countries. The resulting Washington Declaration^6^ established a shared global commitment to advancing exposomics as a scientific discipline, policy priority, and public health imperative. Key outcomes include living labs and citizen science emphasizing community engagement and participatory monitoring, embedded ethics and governance learning from genomics to ensure anticipatory, inclusive, and transparent research, open science and FAIR data with commitment to making tools and findings openly available, and multi-omics AI integration fusing diverse datasets using machine learning to uncover exposure-health links.

#### Relevance to emerging contaminants and environmental exposome

The HEP framework is particularly relevant for addressing challenges in emerging contaminant identification and environmental health assessment. Environmental contaminant discovery involves suspect screening workflows using RT prediction to identify pharmaceuticals, pesticides, and industrial chemicals in environmental samples, transformation product identification for understanding how parent compounds degrade in the environment and biological systems, and exposure pathway mapping to trace contaminant sources from environment to human biomarkers. Non-targeted analysis enhancement includes AI-powered structural elucidation combining MS, RT prediction, and QSAR modeling, database expansion to include environmental chemicals and their metabolites, and cross-platform integration enabling comprehensive chemical coverage across different analytical methods. Real-world exposure assessment encompasses biomonitoring applications to detect environmental contaminants in human biological samples, population-level surveillance for emerging chemicals of concern, and exposure-response modeling to understand dose–response relationships for environmental chemicals.

#### Scientific and economic impact potential

The economic impact of the HEP is projected to match or exceed that of the Human Genome Project. Early indicators suggest significant potential through healthcare cost reduction via prevention-focused approaches, innovation drivers in environmental diagnostics, personalized exposure profiling, and digital health applications, new therapeutic development based on exposure-disease relationships, and regulatory science advancement through improved chemical safety assessment methods. The National Institutes of Health has announced at the Moonshot Forum the development of a Real-World Data Platform that will integrate clinical, genomic, behavioral, and environmental data at scale, with exposome integration as a scientific imperative.

#### Challenges and opportunities

Technical challenges include data integration complexity when combining diverse data types across multiple scales, analytical gaps requiring improved methods to detect and identify unknown chemicals, standardization needs for harmonized protocols for exposure assessment and data sharing, and computational requirements for AI models capable of handling massive, heterogeneous datasets. Societal considerations encompass privacy and ethics in protecting individual exposure data while enabling population health insights, equity and access to ensure global participation and benefit-sharing, public engagement to build community trust and participation in exposome research, and regulatory integration to translate exposome findings into effective public health policies.

#### Future directions

The Human Exposome Project represents a paradigm shift from reactive to preventive approaches in environmental health. Key priorities include methodological development in advancing retention time prediction, AI-driven identification, and multi-omics integration; infrastructure building to establish global networks for data sharing and collaborative research; capacity building to train the next generation of exposome scientists and regulatory scientists; and translation to convert exposome discoveries into actionable public health interventions.

As stated by participants in the Exposome Moonshot Forum: “*If the genome was Apollo 11, the exposome is Artemis—same audacity, bigger destination.*” The integration of advanced analytical methods, including retention time prediction and AI-driven approaches, will be essential for realizing this ambitious vision and addressing the growing challenge of environmental chemical exposures in human health.

#### Conclusion

The Human Exposome Project represents an unprecedented opportunity to understand and address the environmental determinants of chronic disease. By systematically mapping the chemical, physical, and biological exposures that shape human health, this initiative promises to transform our approach to disease prevention and environmental protection. The success of this endeavor depends on continued advancement in analytical methodologies, including the integration of retention time prediction with QSAR and AI approaches described in this review, enabling confident identification of the vast array of environmental chemicals that influence human health throughout the lifespan.

## Metabolite identification in untargeted metabolomics as the bottleneck for a Human Exposome Project

The promise of the HEP is to systematically map environmental exposures and link them to human health outcomes. Untargeted metabolomics, particularly when applied to blood samples, is a cornerstone of this vision (1): it enables comprehensive profiling of endogenous and exogenous small molecules reflective of exposure history. Yet, despite the remarkable sensitivity and coverage of LC–MS and GC–MS, a fundamental bottleneck persists—the structural identification of detected features. Most features in untargeted metabolomics remain unidentified or only tentatively annotated, limiting their interpretability, reproducibility, and utility for regulatory or clinical translation.

This identification bottleneck arises from several factors: the high dimensionality and redundancy of mass spectral features, the overlap of mass-to-charge ratios (m/z) among isomers and analogs, limited MS/MS fragmentation data for all features, and the lack of comprehensive spectral databases that include environmental chemicals and transformation products. These challenges are compounded by the fact that many detected substances are not present in existing libraries (e.g., HMDB, NIST), particularly those of emerging concern such as industrial by-products, metabolites of synthetic chemicals, or food-derived xenobiotics.

Metabolomics as the ‘omics technology closest to phenotype is of critical importance for the future of toxicology (14, 15) and its transition to HEP (16). In deeply phenotyped cohorts, data sparsity is a critical challenge. Llera et al. (17) applied a multivariate imputation framework using Round-Robin regression and Extra Trees to restore missing clinical variables in autism datasets. Such imputation workflows are directly applicable to exposomics, where missing covariates or sample-level measures often limit integrative analyses. LC–MS untargeted workflows face several technical limitations that directly impact exposome research: variable MS ionization efficiencies, retention time drift, and high false discovery rates in compound annotation.

In simple terms, the more comprehensively and accurately we can identify both exogenous substances (those that come from outside the body, such as pollutants, food additives, or drugs) and endogenous metabolites (those produced within the body as part of physiological or pathological processes), the more effectively we can generate meaningful exposure hypotheses linked to health outcomes. Importantly, this must be done in an untargeted fashion—that is, not limited to a predefined list of “usual suspects” or known chemicals of concern.

This has to come on top of the quality assurance, quality control and reporting quality the field of metabolomics needs (15). Starting with our workshop in 2013 (18), a number of QA & QC activities started including our work on peak-calling (19); most notably the Metabolomics Quality Assurance & Quality Control Consortium (MQACC)^7^ published a number relevant articles (20–22). As highlighted in a recent review (23), systematic use of standards and reference materials is essential for ensuring reliability, accessibility, and sustainability of omics-based methods in regulatory toxicology, including LC–MS metabolomics. Notably, generally accepted reporting standards are still missing (24), which would facilitate the integration of RT in the analysis of untargeted metabolomics.

Historically, exposome research has been constrained by a targeted mindset: measuring what we already suspect to be harmful. While this has led to important regulatory actions, it inherently overlooks the vast chemical “dark matter” we are exposed to—unmonitored industrial compounds, transformation products, and low-abundance dietary or environmental exposures that may nonetheless contribute to chronic disease. The untargeted approach enables discovery-driven science: it casts a wide net, allowing us to detect unexpected exposures and their biological effects, even in the absence of prior hypotheses.

High-resolution untargeted metabolomics, when paired with robust annotation pipelines, provides a window into both external exposures and their downstream effects on host metabolism. Each unidentified feature that is correctly assigned a structure potentially represents a new piece of the puzzle: a candidate environmental contributor, a biomarker of past exposure, or a mechanistic link to disease pathways. In this way, high-coverage, high-confidence chemical identification does not merely enhance the data—it expands the landscape of plausible etiological hypotheses, guiding future epidemiology, mechanistic toxicology, and even regulatory prioritization.

The increasing diversity of environmental chemicals entering biological systems necessitates broader analytical coverage (25, 26). Techniques such as LC–MS and GC–MS remain foundational, but real-time platforms like proton-transfer-reaction mass spectrometry (PTR-MS) and selected ion flow tube mass spectrometry (SIFT-MS) offer additional promise for volatile or unstable contaminants, particularly in “breathomics” or ambient exposure contexts. For exposomics to scale, integration of these methods into a shared data backbone—with AI models to unify and interpret output—is a necessary next step (27).

In essence, the better we can characterize the full chemical footprint of human exposure and response—without preconceived filters—the closer we move toward realizing the central goal of the exposome: to map the complex, lifelong environmental influences that shape health and disease.

## Literature search methodology

This is a narrative review based on a corpus of literature collected over the last decade. To ensure comprehensive coverage of recent developments in RT prediction for metabolomics, we conducted a structured literature search informed by systematic review principles. AI tools such as Perplexity.ai, Gemini and ChatGPT were used to identify literature for select aspects, which was accessed and verified.

### Databases and time frame

We searched multiple bibliographic databases including PubMed, Scopus, and Google Scholar for relevant literature published up to July 2025, in order to capture the rapid methodological progress of recent years (2023–2025). Articles from early 2025, including select preprints, were considered when peer-reviewed alternatives were not yet available. In addition, using elicit.ai, a search of 126 million abstracts from the Semantic Scholar corpus was carried out with the prompt “*What are advances in the automated identification of metabolites in untargeted metabolomics?* “This retrieved 493 papers most relevant to the query, which were screened for these criteria: Computational Method Focus: Does the study present computational methods or algorithms for metabolite identification in untargeted metabolomics data? Analytical Platform: Does the study use mass spectrometry (MS) data? Method Innovation: Does the study present either a novel approach or significant improvements to existing automated identification methods? Method Validation: Does the research include validation of the automated identification methods? Metabolomics Approach: Does the study include untargeted metabolomics analysis (not exclusively targeted)? Annotation Method: Does the study include automated (not exclusively manual) annotation methods? Computational Component: Does the study include computational components beyond pure analytical chemistry? This resulted in 40 studies with focus: Identification was the most common focus, found in 19 studies; Annotation was the focus in 13 studies; Feature detection was addressed in 6 studies; Classification was the focus in 2 studies; Benchmarking was the focus in 1 study; some studies addressed more than one focus. These were further analyzed by Elicit.ai, to extract information, which was not used because of some obvious mistakes, but the downloaded papers were added to our review corpus.

### Search terms and strategy

Representative search strings included: “retention time prediction metabolomics,” “QSR model LC–MS,” “retention index GC metabolomics,” “machine learning retention time,” “MetFrag RT scoring,” and “SIRIUS CSI FingerID retention.” We combined terms for chromatographic modes (reversed-phase, HILIC, GC) with those for prediction or algorithms (e.g., deep learning, GNN, transfer learning). Author names associated with seminal works (e.g., Kaliszan, Héberger, Ruttkies) were also used as search terms to retrieve foundational and follow-up contributions. Some references from within the papers identified, were also retrieved and added to the review corpus.

### Inclusion and exclusion criteria

We included publications that: (i) introduced new RT prediction models; (ii) reported large RT datasets; or (iii) demonstrated integration of RT information into metabolite identification workflows. Both methods-focused studies (e.g., model development, algorithm benchmarking) and application studies (using RT for exposomics identifications) were incorporated to balance theory and practice. Key review articles [e.g., (6, 7)] were also cited to ensure coverage of foundational principles. Exclusion criteria encompassed papers that were overly narrow in scope (e.g., QSRR models limited to a single small chemical class), those lacking sufficient methodological data, or those only mentioning RT without substantive evaluation. Non-English articles were excluded, and preprints were only cited when indispensable.

### Bias control

To minimize bias, we deliberately sought coverage across all major chromatographic modalities (RP, HILIC, GC) and across both classical (MLR, SVR, RF) and modern AI approaches (DNNs, GNNs, transfer/meta-learning). Multiple independent studies were cited for each major conclusion whenever possible—for example, drawing on results from different groups to validate reported error ranges in HILIC prediction. Findings were cross-checked for consistency across studies to avoid over-reliance on single laboratories. Furthermore, limitations and failure modes of RT prediction models are discussed explicitly in the manuscript to prevent over-optimistic interpretation.

Although this review is narrative in scope, the inclusion of this methodology section is intended to increase transparency, demonstrate rigor in source selection, and provide guidance for readers seeking to replicate or extend the literature survey.

## Role of retention time in metabolite identification

RT is the time a compound spends in the chromatographic column before detection. It encodes information about the compound’s interactions with the stationary phase and mobile phase. Compounds with different structures typically have different RTs, especially under well-controlled conditions. Thus, RT provides an additional dimension for identification beyond mass. For example, among candidates with the same m/z, those with predicted RTs incompatible with the observed RT can be eliminated. Using RT as a filter can significantly narrow down candidate lists, focusing on structures whose properties match the chromatographic behavior. This is particularly useful when fragmentation data are limited or absent. In many untargeted studies (e.g., pilot studies or those with limited sample), MS/MS spectra may not be available for every feature. In such cases, analysts must rely on m/z and RT alone, which yields a putative identification (below the Metabolomics Society identification (MSI) confidence of MSI Level 2, as Level 2 formally requires MS/MS spectral confirmation). Incorporating RT predictions can nonetheless raise confidence in these tentative IDs by requiring consistency between predicted and observed RT.

However, raw RT values are not directly comparable across experiments without correction. Even the same compound can have different RTs on different systems or on the same system over time. This lack of reproducibility historically limited RT’s usefulness in databases. Calibration and standardized retention indices (RI) are solutions to make RT more transferable. In GC–MS, Kovats retention indices (using alkane standards) have long been used to match experimental RTs to library values, greatly aiding identification. By contrast, LC–MS lacks a universally adopted retention index system—as noted in early studies, “*no such index currently exists for LC–MS experiments*” and run-to-run RT variation complicates direct comparison (28). They recommend robust RT alignment, pooled QC analysis, and multidimensional chromatography to address these challenges. Retention time prediction has also proven valuable for distinguishing structural isomers, which are otherwise indistinguishable by accurate mass alone. In a forensic toxicology context, Tyrkkö et al. (29) used ACD/ChromGenius to correctly predict elution order for over two-thirds of isomer groups, underscoring the utility of RT as a structural discriminator even in complex matrices. In the following sections we explore how new methods predict RT from structure and calibrate RT across systems to overcome these issues.

## Retention time behavior across chromatographic modalities

Different chromatographic modalities separate molecules based on different physicochemical interactions, so the retention time meaningfully reflects different properties in each modality. A comprehensive RT-based identification approach must therefore account for the specific chromatography used, which include reversed-phase (RP) LC; hydrophilic interaction LC (HILIC), ion-exchange and ion-pair LC as well as gas chromatography (GC). Table 1 summarizes key datasets (e.g., SMRT, RepoRT) and representative model performances across modalities [RP, HILIC, GC Retention Index (GC/RI)]: N (compounds), gradient window, train/test split, MAE/median error, and external-method transfer results.

**Table 1:** Benchmarking of retention time prediction models and datasets across modalities.

Dataset/study	Chromatographic mode	Size (*N*, compounds)	Gradient/RI window	Model type (s)	Performance (MAE/median error, transferability)
METLIN SMRT (4)	RP-LC (C_18_, 10 min)	~80,000	0–10 min	DNN, Gradient Boosting	MAE ~ 39 s; median ~17 s (~5% RT); robust cross-validation
RepoRT (41)	RP-LC + HILIC, 49 methods	8,809 compounds (88,325 RTs)	Varied (2–30 min gradients)	Graph Neural Networks (GNN), Transfer Learning	Error ~0.3–1.8% of RT; post-calibration <0.15 min across platforms
HILIC QSRR (33, 34)	HILIC (polar stationary phase)	100 s–1,500	10–20 min	Random Forest, Linear QSRR	*R*^2^ up to 0.97; eliminated ~40% false positives in annotation
NIST retention index DB (GC)	GC–MS, Kovats RI scale	~180,000	RI scale (C8–C40 n-alkanes)	DL, SVM, RF	Median error ~20–40 RI units (~0.5–1% RI scale); correct ID first-rank in up to 86%
EndoRI (42)	RP-UPLC (endo-calibrants, acylcarnitines)	~200–300 standards + endogenous calibrants	0–12 min	Linear calibration, meta-learning	Reduced inter-batch RT variability by 95% (1.5 → 0.15 min)

RPLC datasets (e.g., METLIN SMRT, RepoRT) underpin modern deep learning approaches, achieving approximately seconds-level prediction accuracy. HILIC (hydrophilic interaction liquid chromatography) datasets are smaller but show strong relative retention time (%RT) prediction performance when models are trained specifically for polar chemistries. GC datasets (Retention Index, RI) benefit from highly standardized conditions, yielding very low relative errors (~1% of RI scale). Calibration strategies (endoRI, transfer learning) dramatically improve cross-platform transferability.

### Reversed-phase (RP) LC

This is the most common modality in metabolomics (e.g., using C_18_ columns with aqueous/polar organic mobile phases). Retention depends on partitioning between the stationary and mobile phase, and increases with hydrophobicity. Thus, in RP-LC, RT correlates strongly with measures like octanol–water partition coefficient (logP), number of nonpolar carbons, and aromaticity. QSRR models for RP often use descriptors capturing hydrophobic surface area, H-bond donors/acceptors, etc., which relate to logP. Indeed, a simple linear relationship between logP and RT is sometimes used for rough predictions or to filter candidates. Tools like MetFrag^8^ exploit this: given a set of reference compounds with known RT, MetFrag can derive a linear model RT = a·(predicted logP) + b, and then score candidates by how well their predicted logP fits the observed RT. MetFrag2.2 (30) represents a significant evolution in *in silico* annotation, integrating structure-based fragmentation with orthogonal scoring layers such as predicted RT, database references, and user-defined filters. These functions make MetFrag2.2 a foundational component of high-throughput in many exposomic workflows. More advanced RP models use nonlinear ML to capture subtle effects (e.g., polar groups that cause shorter RT than logP alone would suggest). Since the METLIN SMRT dataset is RP-based, most of the recent deep learning models (DNNs, transformers, etc.) have focused on RP-LC predictions. The accuracy achieved (median errors on the order of seconds) shows that RP retention can be predicted very effectively across diverse chemical classes. However, note that extremely polar or ionic compounds may be “non-retained” (elute in the void volume) on RP; these need special treatment (e.g., classification models to predict if a compound will be non-retained). Aalizadeh et al. (31) developed QSRR models for thousands of emerging contaminants across both RPLC and HILIC platforms and introduced OTrAMS and MCS to map error distributions and define prediction applicability domains. This combination improves confidence in suspect screening by reducing false positives and providing formalized acceptance windows for predicted RTs.

### Hydrophilic interaction LC (HILIC)

HILIC, a hybrid technology between normal phase LC (NP-LC) and RP-LC, uses a polar stationary phase, which would be common in NP-LC, and combines it with a polar mobile phase containing solvents common in RP-LC, such as acetonitrile or methanol. It retains very polar and hydrophilic compounds that are poorly retained in RP. Retention in HILIC tends to increase with polarity and the ability to form hydrogen bonds—essentially the opposite trend of RP. Properties like polar surface area, number of charged or polar functional groups, and dipole moments become important. HILIC retention prediction is inherently more complex in some cases due to possible multiple interaction mechanisms (partitioning and adsorption). Nonetheless, researchers have developed HILIC-specific models. For instance, versions of graph neural networks have been trained for HILIC retention (“GNN-TL-HILIC”) distinct from RP models. The availability of HILIC data is more limited than RP, but growing. A recent report noted successful RT prediction for >1,500 metabolites on 24 different LC systems including HILIC, with median errors ~0.3–1.8% of the RT (32). This suggests that with proper training, HILIC RT can also be predicted and used for identification—albeit one may need separate models or transfer learning from an RP model. When annotating unknowns in HILIC, one should use a model trained or calibrated for HILIC to avoid systematic bias. In HILIC chromatography, where retention behavior is highly nonlinear and condition-sensitive, Cao et al. (33) achieved a Pearson correlation of 0.97 between predicted and experimental RTs using a Random Forest QSRR model. This level of precision allowed systematic reduction of false positives during peak annotation, even without MS/MS confirmation. In a foundational study, Creek et al. (34) developed a QSRR model for HILIC chromatography and demonstrated that RT prediction improved annotation precision, eliminating 40% of false positives from exact-mass matches alone. This underscores the importance of orthogonal RT filtering for increasing annotation confidence in metabolomics. The approach by Karlberg et al. (35) to predict hydrophobic interaction chromatography (HIC) retention of monoclonal antibodies using sequence-, model-, and dynamics-derived descriptors offers a useful analogy for metabolite RT modeling. Their success with 3D descriptors suggests that deep molecular representations may enhance QSRR model performance in highly diverse exposomic datasets.

### Ion-exchange and ion-pair LC

These modalities separate compounds based on charge interactions. In ion-exchange chromatography, a charged stationary phase (cation or anion exchanger) retains oppositely charged analytes. Retention depends on the ionic strength, the charge of the molecule (which in turn depends on pH and pKa), and how strongly that charge interacts with the stationary phase. For metabolomics, ion-exchange is less commonly used in untargeted profiling (since it is highly selective), but it can appear in targeted methods (e.g., analysis of amino acids or organic acids). Ion-pair LC is a variant of RP where an ionic reagent in the mobile phase forms ion pairs with analytes, effectively allowing charged molecules to be retained, for example, on a C_18_ column. Predicting retention in such systems requires understanding of both hydrophobic and ionic characteristics. QSAR models might include descriptors for pKa, net charge at the working pH, and interactions with counter-ions. While fewer public studies exist on machine learning for ion-exchange LC, conceptually one could train a model if a dataset of retention times for charged metabolites (with a given ion-pair reagent or ion-exchange column) is available (36). Calibration is particularly important here: small changes in mobile phase pH or salt can shift RT substantially. Including internal standards of known pKa/charge can help model the retention. In summary, while ion-exchange modes are not as prevalent in untargeted workflows, the same principles apply – one would need a method-specific retention model, and using RT for identification in these modes works best when the unknown and references share similar ionic properties.

### Gas chromatography (GC)

GC separates compounds based on volatility, vapor pressure and partitioning between the stationary phase and carrier gas (often correlating with boiling point and hydrophobicity). In metabolomics, GC–MS (usually with electron ionization, EI) is commonly used for volatile metabolites and requires derivatization for polar compounds. Retention indices (RI) in GC are well-established for compound identification. Libraries like NIST and Wiley contain thousands of spectra with associated retention indices on standard columns (e.g., DB-5 or polar columns), allowing dual matching: spectral pattern and RI. In unknown identification, if the experimental RI of a feature matches a library compound’s RI within a tolerance (e.g., ±10 index units) and the spectra match, the identification is considered confident. RT prediction in GC can leverage the vast RI libraries. Researchers have applied deep learning to predict Kovats retention indices: for example, a study by de Cripan et al. (39) demonstrated deep learning (DL) prediction of RI on polar and mid-polar columns, marking the first use of DL for GC retention (37). Another study focused on trimethylsilylated metabolites (common in GC derivatization) built an SVM-based model that predicted RI with ~37 units median error (40). They showed that using predicted RI to rank candidates could reliably identify the correct structure among isomers. The advantage in GC is that, thanks to RI calibration with standards (n-alkanes), the predicted RI can be directly compared to literature/library values, which are highly reproducible (unlike absolute RT in LC). Thus, integrating AI-predicted RI with GC–MS libraries can flag which candidate is most plausible. For instance, if an unknown’s EI spectrum matches several compounds but their literature RIs differ, one can compute which candidate’s RI is closest to the observed. In practice, GC identification already uses retention as a key factor; AI simply enhances this by predicting RI for compounds not yet experimentally measured. As more metabolite RI data become available (e.g., via the MetaboLights (38), FiehnLib,^9^ or HMDB^10^ GC libraries), we anticipate even better predictive models.

In summary, each chromatographic modality requires a tailored approach to RT prediction. The fundamental strategy remains: train or apply a model appropriate to that modality, and calibrate if necessary, using reference compounds. RPLC and GC are currently the most mature in terms of available data and models, but HILIC and others are catching up. An ideal pipeline in untargeted metabolomics might involve running samples on multiple platforms (e.g., RPLC- and HILIC-MS, plus GC–MS for volatile fraction) and using RT predictions in each domain to aid identifications. This would cover a wide swath of metabolome polarity and use RT information optimally in all cases.

## Retention time prediction (QSRR) for candidate ranking

Retention time prediction models (QSRR) use molecular descriptors or structural fingerprints to predict a compound’s RT on a given chromatographic system. These are essentially quantitative structure–retention relationship models, akin to QSAR models for activity or other properties. By training on compounds with known structures and RTs, a model can learn the relationship between molecular features (e.g., hydrophobicity, polarity, functional groups) and retention. The model can then predict RT for new compounds and thereby assist identification (4, 6, 7, 30, 40, 48). In practice, one would take the list of candidate structures (proposed by matching m/z or MS/MS to databases) and rank or filter them by comparing predicted RT to the observed RT of the unknown feature. Candidates whose predicted RT deviates greatly from the experimental RT are less likely to be correct.

Studies have demonstrated that RT-based ranking significantly improves the chance of including the correct structure among top hits. For example, García et al. (4) trained machine learning regressors (including deep neural networks and gradient boosting) on ~80,000 compounds’ RTs (the METLIN SMRT dataset) for a reversed-phase LC system. They integrated these predictions into a metabolite annotation workflow, assigning a probabilistic score (*z*-score) based on how well a candidate’s predicted RT matches the observed RT after accounting for prediction uncertainty. In a test where candidates were filtered by mass and then ranked by this RT score, the correct molecule was among the top 3 candidates in 68% of cases—a substantial improvement over mass-based ranking alone, where baseline values were around 51–60% depending on the dataset. This highlights that using RT as an additional criterion can greatly enhance identification success. Similarly, in GC–MS, where RT indices are routinely used, a recent machine learning model predicted retention indices for trimethylsilyl-derivatized metabolites with median error ~37 index units, and using these predictions to rank candidates placed the true identity first in up to 86.7% of cases with two candidates (and significantly improved top-3 ranking for multiple candidates) (39). Although RT predictions may not perfectly eliminate all false hits, they *prioritize* the most plausible structures. Indeed, one study found that the ranking power of predicted RT was comparable to that of MS/MS spectral matching—combining both yields the best results (28).

It is important that RT predictions have to be reasonably accurate (on the order of a few seconds to at most a minute error for LC, or a few RI units for GC) to be useful. Early QSRR models suffered from limited accuracy due to small training sets (hundreds of compounds). This typically restricted models to narrow chemical classes or yielded only rough retention order predictions. The situation has improved dramatically with larger datasets. The METLIN SMRT dataset release (80 k compounds) “renewed interest in RT prediction” and enabled general models covering diverse small molecules. Modern ML models now achieve mean absolute errors on the order of seconds: for example, evaluating performance using the Mean Absolute Error (MAE) and the Median Absolute Error (MEDAE), both reported in seconds, the best model by García et al. (4) had ~39.2 ± 1.2 s mean error (17.2 s median) on a ~ 10-min gradient, which is typically within ~5% of RT. Another advanced model (RT-Transformer, 2024) achieved ~27–33 s error in tests, and importantly maintained accuracy across different chromatographic methods via transfer learning (40). Such accuracy is sufficient to discriminate many isomers or to flag candidates with clearly mismatched RT. In practical workflows, one would usually set an RT tolerance window or score function. For instance, if an unknown elutes at 5.00 min, and one candidate is predicted at 4.9 min while another at 8.0 min, the first will score much higher. Inclusion of RT scores in tools like MetFrag has shown drastic improvements: Ruttkies et al. (30) reported that adding retention information (along with other metadata) improved the top-rank identification rate from only ~6–9% (using mass and MS/MS alone) to 71–89% when RT and reference data were considered. Clearly, QSRR models provide a powerful filter to reduce false positives in annotation.

Fitch et al. (41) proposed a standardizable Chromatographic Hydrophobicity Index (CHI) and CHIbt, a standardized RT-shift descriptor for interpreting Phase I drug biotransformations. By linking CHI to physicochemical descriptors like clogP and hydrogen bond donors, they enabled metabolite annotation using predicted RT behavior—a strategy that could be adapted to untargeted xenobiotic exposomics.

## Calibration of retention time and retention indices

**Figure 1:**
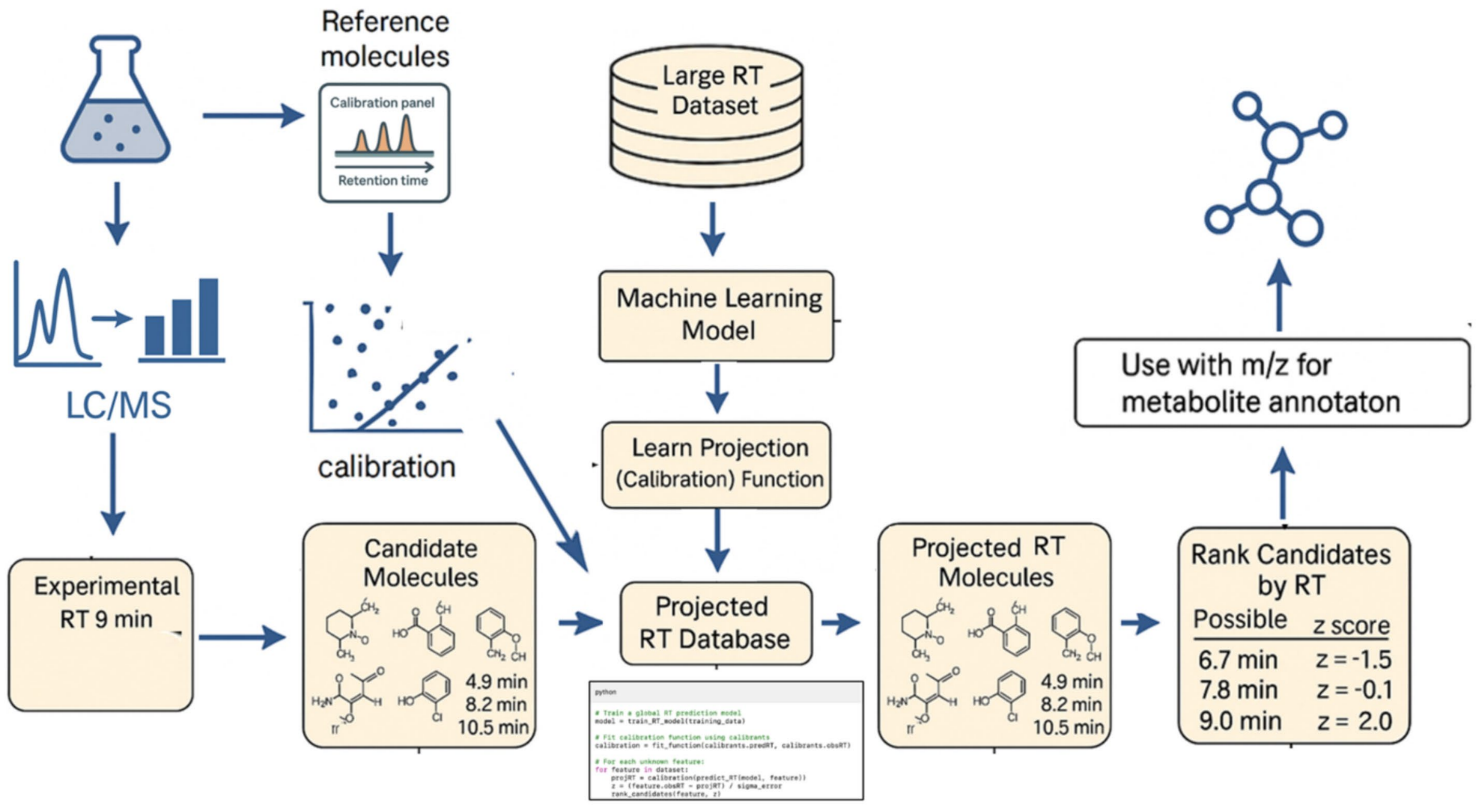
Illustrative workflow for integrating retention time prediction and calibration into metabolite annotation. To start, a machine learning model is trained on a large RT dataset. From these, a projection (calibration) function is learned to map RTs to given LC or GC measurement conditions. A small number of known reference molecules in the sample (with known identities and RTs, e.g., human blood metabolites as discussed later) are used to calibrate experimental vs. predicted RT. The model’s expected RT for all candidate structures is adjusted to the current chromatographic conditions, yielding a “projected RT.” For an unknown feature, its experimental RT is compared to the projected RTs to find matching candidates, and candidates are ranked by how close their RT is (accounting for uncertainty via a *z*-score). This approach allows retention time to be used alongside m/z for more accurate metabolite annotation.

One of the main hurdles in using RT broadly is variability: RT can shift due to instrument differences, column aging, or subtle changes in mobile phase or temperature. Therefore, calibration strategies are essential to correct RT shifts and make predicted RT applicable across different runs and machines. Calibration can be achieved by using well-characterized reference substances—either spiked standards or endogenous compounds—with known retention behavior. The idea is to adjust the RT scale of an experiment to a reference scale (or model) via a mapping function (Figure 1).

A simple calibration approach is the use of retention index (RI) systems, analogous to GC. In GC–MS, a series of n-alkane standards is often run, and each compound’s RT is converted to an index (e.g., Kovats index) relative to those standards. This compensates for differences in temperature ramp or column length. For LC, various RI schemes have been proposed. For example, the chromatographic hydrophobicity index (CHI) uses a set of standard compounds to create a reproducible scale for reversed-phase HPLC. More recently, researchers have explored using endogenous metabolites as calibrants. Chen et al. (42) developed an “endogenous retention index” (endoRI) method for ‘RP ultra performance liquid chromatography (RP-UPLC). By leveraging straight-chain acylcarnitines present in biological samples as internal calibrants. Since acylcarnitines (C_2_ to C_18_, etc.) are naturally found in plasma/serum, they provide a built-in homologous series. The team established a quantitative relationship between acylcarnitine chain length and RT, and used this to correct RTs in each run. The result was a dramatic reduction of RT variability: inter-batch and inter-platform RT differences dropped from ~1.5 min to 0.15 min (95% reduction) for 95% of metabolites after endoRI correction. In other words, using internal compounds to normalize the RT axis allowed data from different days or instruments to be directly compared with only a few seconds discrepancy.

Retention time prediction using support vector regression (SVR) and random forests trained on 3D molecular interaction field (MIF)-based descriptors has shown high utility in identifying unknowns in untargeted UPLC–MS workflows. In a study using over 400 authentic standards, Wolfer et al. (43) demonstrated >80% reduction in false positives and effective applicability domain modeling via self-organizing maps (SOMs).

More generally, calibration can use any set of known compounds spanning the RT range of interest. In practice, one might include a mix of standards in each run, or rely on identified endogenous metabolites, to act as anchors. These anchors are run through the same LC–MS method and their known identities allow one to either: (a) adjust the RT prediction model (e.g., via linear regression, bias correction, or more complex projection) or (b) create an index. Approaches like the Bayesian meta-learning projection by García et al. (4) require as few as 10 known molecules in an experiment to calibrate predictions to that specific method. The model uses the small set of observed RTs to learn a mapping (with uncertainty estimates) from predicted RT to actual RT for that method. Once calibrated, the predicted RTs are converted to “projected” RTs for that run, which can be directly compared to experimental values. This not only accounts for systematic shifts (e.g., all compounds eluting 0.5 min later than predicted) but can also handle differences in gradient length or column chemistry to some extent. The use of *z*-scores or confidence intervals, as implemented by García et al. (4), means that the ranking considers prediction uncertainty—a candidate whose projected RT falls well within the experimental RT error band will score higher than one at the edge of the range.

Best results are often achieved by multi-point calibration covering the chromatographic range. A recent comprehensive study by Zhou et al. (28) evaluated cross-method RT projection using 30 different LC methods and 330 compounds, with various sets of calibrants. They found that using 30–70 carefully selected calibrants that span different retention behaviors allowed accurate projection between very different chromatographic setups. When source and target methods were similar (e.g., same C_18_ column and mobile phase), projected RTs had <0.5 min error (<3% of gradient). Even between quite different methods, errors were mostly within ~2 min after calibration. This underscores that with a sufficient and representative calibrant set, one can translate RT predictions from one modality to another with high fidelity.

A recent breakthrough in LC–MS retention alignment is the development of a system-agnostic RI, proposed by Aalizadeh et al. (31), which allows mapping RT across labs and platforms using 18 carefully selected calibrants. This harmonization makes retention time a transportable identifier, analogous to Kovats indices in GC. The RI approach holds particular promise for multi-cohort exposomic studies and should be evaluated in blood-based untargeted workflows.

In summary, calibration strategies—whether using a fixed index scale or adaptive modeling with internal standards—are crucial to unlock RT’s full potential for unknown identification. We recommend that analysts include a set of known metabolites (endogenous or spiked) in each batch as RT calibrants. Notably, human blood has many well-characterized metabolites (glucose, amino acids, lipids, etc.) that can serve this role without needing exotic standards. By modeling RT against these references, labs can correct drift and even share comparable RT data across instruments. This makes retention time a robust, transferable identifier rather than a lab-specific observation.

## AI and machine learning for RT and physicochemical property prediction

Recent years have seen rapid growth in AI and machine learning methods for predicting retention time and related physicochemical properties. Traditional QSRR approaches used linear regressions or simple machine learning (like partial least squares or random forests) on computed molecular descriptors. Artificial neural networks (ANNs) have shown promise for RT prediction across large and diverse chemical sets. Bade et al. (44) trained an ANN that achieved 95% RT prediction accuracy within 2 min, enabling high-confidence screening of drug metabolites and transformation products in wastewater without reference standards. Modern approaches increasingly leverage deep learning to automatically learn features from molecular structures, often outperforming descriptor-based models. Heinonen et al. (45) pioneered a two-step ML method (FingerID) to predict molecular fingerprints from MS/MS spectra and match them to PubChem structures, enabling *de novo* metabolite annotation even in the absence of reference spectra. This framework underpins many modern identification algorithms and supports discovery beyond known databases. In a comprehensive benchmarking study, Bouwmeester et al. (46) evaluated seven ML algorithms for retention time prediction across 36 LC–MS datasets. While no single method outperformed others across all conditions, gradient boosting consistently ranked among the top performers with minimal overfitting risk. Their findings underscore the need for tailored algorithm selection or model blending to optimize RT prediction in diverse exposomic contexts. Below we highlight key advancements in deep neural networks, graph neural networks, transfer learning and meta-learning, physicochemical property prediction, and other AI applications in identification.

### Deep neural networks (DNNs)

Multilayer feed-forward neural nets can model complex nonlinear relationships between structural features and RT. García et al. (4) used a deep neural network (with advanced training tricks like cosine annealing and weight averaging) to achieve state-of-the-art accuracy on the SMRT dataset. The DNN outperformed other regressors, indicating that enough training data can unlock the predictive power of deep learning. DNNs treat molecular descriptors or fingerprints as input; one challenge is deciding how to represent the molecule. In García’s work, they provided over 5,000 molecular descriptors (alvaDesc) plus 2,214 binary fingerprints as input features. The network then learned which features correlate with retention. The result was a median error of ~17 s, which was (at the time) the most accurate published result for small-molecule RT prediction. This demonstrates that with enough data, a neural network can implicitly learn classical retention trends (like hydrophobicity) as well as more subtle structural effects (ring systems, functional group interactions with the column, etc.). García et al. (4) introduced a probabilistic annotation framework that combines deep learning-based RT prediction with Bayesian meta-learning for method-specific projection. Their approach converts predicted RTs into probabilistic z-scores relative to a small set of identified metabolites, enabling accurate ranking of annotation candidates even across different chromatographic systems. In GC–MS-based metabolomics, de Cripan et al. (39) showed that accurate retention index prediction of trimethylsilyl (TMS) derivatives can be achieved using support vector machines and Dragon molecular fingerprints. They further demonstrated that prediction accuracy correlates strongly with the Tanimoto similarity of training-test structures, providing a quantitative confidence estimate for each RI prediction. In lipidomics, where chromatographic behavior varies due to matrix complexity and compound polarity, Noreldeen (47) developed a robust RT prediction model based on molecular descriptors and validated across human and mouse datasets. Crucially, the study demonstrated successful RT calibration across LC–MS instruments using a linear transformation equation, enabling direct reuse of RT-annotated libraries across systems. Matyushin et al. (37) applied deep learning to GC retention index prediction across polar and mid-polar stationary phases, achieving MAEs as low as 16 RI units. Their layered architecture supports extension to 2D-GC applications, making it highly suitable for exposome workflows reliant on derivatized compounds. A comprehensive review by Liu et al. (5) highlighted the surge in deep learning RT models following the release of SMRT (48) and RepoRT datasets, emphasizing transferability, representation artifacts, and the need for curated multi-CM training data. Their summary affirms the trend toward harmonized RT-informed annotation pipelines across experimental conditions.

### Graph Neural Networks (GNNs)

**Figure 2:**
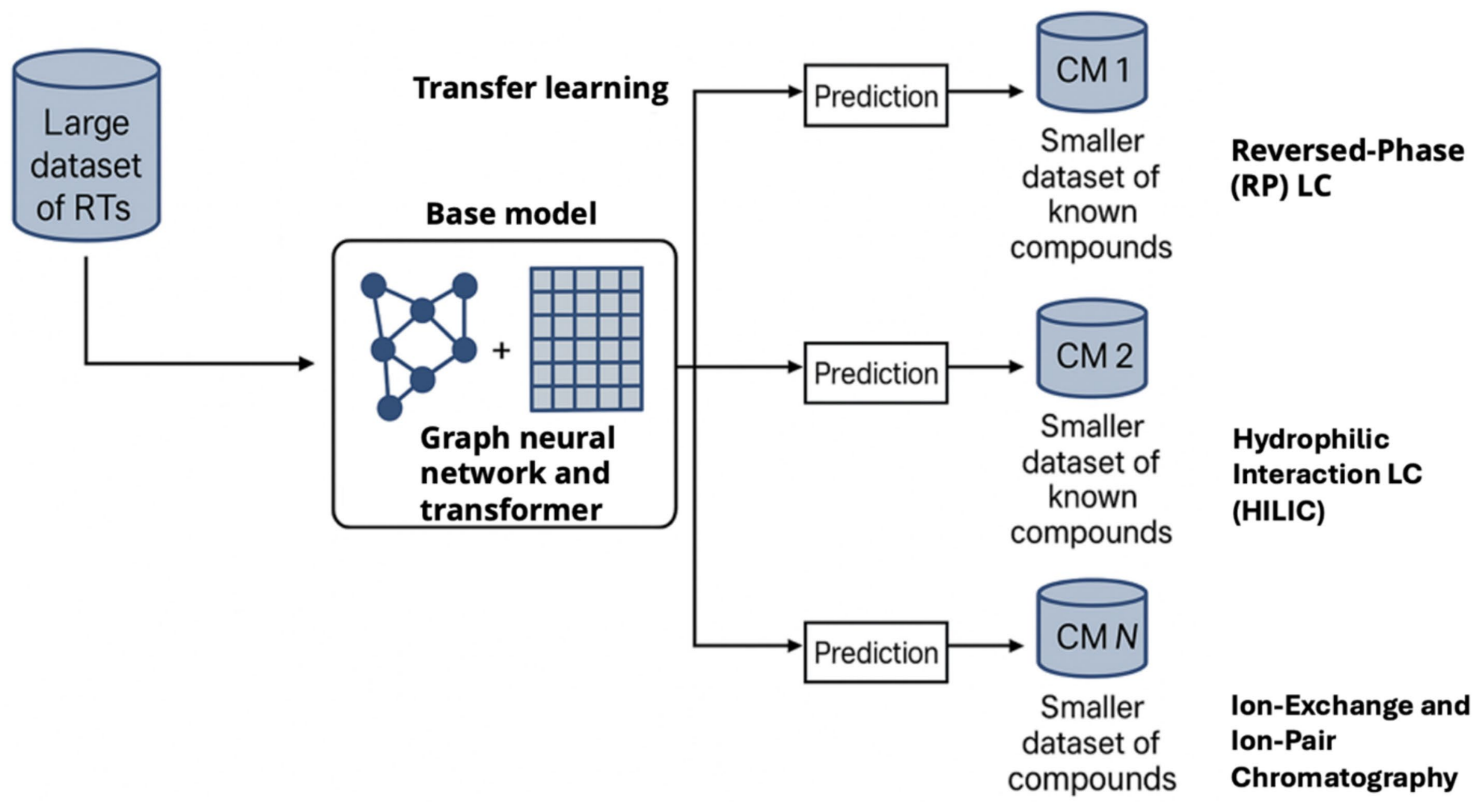
Overview of the RT-transformer deep learning approach for retention time prediction across chromatographic methods. A large dataset of RTs (SMRT, reversed-phase etc.) is used to train a base model (graph neural network + transformer). Through transfer learning, this model can be fine-tuned on new chromatographic methods (CM 1, 2, … *N*) using smaller datasets of known compounds. The resulting model predicts retention times for the specific method with high accuracy. This approach greatly improves scalability and allows retention prediction to assist metabolite identification under varied conditions, as demonstrated by improved annotation accuracy when incorporating predicted RTs.

Instead of relying on pre-computed descriptors, GNNs operate directly on molecular graphs (atoms as nodes, bonds as edges). They perform message-passing to learn an embedding that captures the molecule’s structure. GNNs have shown excellent performance in many chemistry tasks and are naturally suited to generalize to novel structures. For RT prediction, various GNN architectures have been tested. Kwon et al. (60) introduced a graph convolution model that achieved good RT predictions by learning from molecular graphs and was later extended with transfer learning for HILIC vs. RP differences. One advantage of GNNs is that they can inherently capture structural isomer differences that might be lost in simple descriptors. For example, two isomers with the same formula can have different RT; a GNN can be trained to distinguish those by subtle structural cues (like branching versus linear structure affecting hydrophobic surface area). Some models combine GNNs with other deep learning components – Retentive Time Transformer (RT-Transformer) (Figure 2) is a notable example combining a graph attention network (to encode structure) with a transformer network (to encode learned fingerprints in sequence). Xue et al. (38) introduced RT-Transformer, a state-of-the-art hybrid deep learning model combining graph attention networks with 1D-transformers to predict RT across diverse chromatographic systems. Pre-trained on over 80,000 molecules, the model supports transfer learning and achieves a mean absolute error of 27.3 s on external datasets. Its scalability and accessibility make it a valuable tool for RT-informed metabolite annotation in exposomics. This hybrid model was pre-trained on the large SMRT dataset and then fine-tuned on specific chromatographic conditions via transfer learning. The result is a highly flexible model that can “adapt” to any LC method given a small fine-tuning dataset. RT-Transformer achieved competitive accuracy and demonstrated excellent scalability, i.e., the same model architecture can handle RP, HILIC, or other gradients with minimal loss of accuracy. This is a significant advancement because it means labs could leverage a *pre-trained RT predictor* and just calibrate it with a few dozen known compounds for their custom method, rather than needing to train from scratch. It essentially automates the projection concept with deep learning.

### Transfer learning and meta-learning

As mentioned, models like RT-Transformer explicitly use transfer learning to handle different methods. Another approach is meta-learning, where the model is trained to quickly adapt to new tasks. García et al.’s (4) Bayesian meta-learning can be seen as a lightweight version: it learns how to adjust predictions with few samples. In both cases, the underlying principle is that retention mechanisms share commonalities (e.g., a very polar compound will likely elute early in RP, and perhaps later in HILIC—the model can learn such patterns) and differences (the scale and exact ordering differ by method). AI models that can leverage large, pooled data but specialize to local conditions are crucial for practical use, since each lab’s method has quirks.

### Physicochemical property prediction

Besides direct RT prediction, AI is used to predict intermediate properties that correlate with RT. For instance, log P (octanol–water partition coefficient) can be predicted from structure using machine learning. SIRIUS’s interface uses a computed XLogP to filter candidates by expected RT range.^11^ Abrahamsson et al. (49) recently proposed a novel approach that integrates measurements of equilibrium partition ratios between different organic solvents and water (K_SW_) to predictions of molecular structures. This information can be used as a fingerprint and, using machine learning, converted into a series of functional groups that can be used to search chemical databases. Another property is pKa—knowing a molecule’s pKa can help predict if it will be ionized under LC conditions, which drastically affects retention (especially for ion-exchange or if buffers cause partial ionization). Tools like ACD Labs or open-source models can predict pKa, and this information could be fed into retention models or used qualitatively (e.g., expecting that a molecule that is highly ionized might elute early on RP). Deep learning has been applied to many such properties (logP, pKa, solubility, etc.), often achieving better accuracy than older methods. The integration of these predictions in metabolite ID is an emerging area. For example, one could imagine using predicted pKa to choose the correct isomer of an organic acid that matches an ion-exchange retention time. In practice, however, since we can now often predict RT directly, the need for intermediate property prediction is reduced—the model implicitly accounts for them. Still, having accurate property predictors can aid understanding: if a candidate has a predicted logP of 1 but requires logP ~4 to elute at 10 min in a given RP method, that is a quick sanity check to discard it.

### Other AI applications in identification

Beyond RT, AI is heavily used in other aspects of metabolomics. CSI:FingerID (50) uses kernel-SVM methods to predict a molecular fingerprint from MS/MS spectra, which is then compared to database structures. This is an example of ML improving structure ranking by spectral features. CSI:FingerID transformed database searching by inferring molecular fingerprints from MS/MS spectra using supervised machine learning. This allowed metabolite annotation beyond spectrum libraries and became foundational for follow-on tools like SIRIUS and COSMIC. Deep learning has also been used to generate in-silico spectra (to expand MS/MS libraries) and even to propose novel structures from spectra [e.g., MSNovelist (51), MassGenie (52)]. These advances complement RT prediction; a future AI-driven identification pipeline might predict both the spectrum and retention of candidate structures and score everything together against the observed data. Some research already heads this direction, combining multi-modal prediction to boost confidence. As of 2024, the field recognizes that multi-parameter scoring (m/z, MS/MS, RT, even ion mobility CCS) is the way to break the bottleneck of unknown identification. AI provides the tools to predict each parameter with associated confidence, enabling a more holistic comparison between an unknown and candidate structures. The KGMN approach by Zhou et al. (28) unites three network layers-reaction pathways, MS2 similarity, and chromatographic coelution-to propagate annotations from knowns to unknowns recursively. This hybrid model outperforms MS2-only methods by recognizing in-source fragments and prioritizing biochemically plausible transformations.

In summary, AI and machine learning have become indispensable for retention time prediction. They offer accuracy and generalization far beyond what earlier QSAR models could achieve. By deploying deep learning models (either pre-trained or trained on in-house data), researchers can routinely obtain RT predictions to within a few seconds or a few percent error. These predictions, when properly calibrated and combined with other evidence, significantly enhance the reliability of metabolite identification. As datasets continue to grow (e.g., community-driven sharing of RT data) and models improve, we expect retention prediction to become even more robust, possibly incorporating explainability (so one can rationalize why a compound is predicted to elute later—e.g., “due to having a long alkyl chain,” etc.). The key takeaway is that ML-predicted RT is now a viable, validated tool for metabolomics, and its adoption is accelerating.

## Tools and databases for integrated identification

AI critically depends on the availability of Big Data. Several software tools and databases can facilitate the integration of RT predictions with traditional MS-based identification. Table 2 shows a number of these tools and databases.

**Table 2:** Software tools and databases for integrated retention time (RT) predictions.

Database	Owner/institution	Purpose/focus	Public/non-public	Number of samples/spectra	Number of chemicals identified
MetaboLights	European Bioinformatics Institute (EMBL-EBI)	Repository for metabolomics experiments and derived information	Public	270,403 samples, 439,537 data files	1,687,165 metabolites/unknowns/features
Metabolomics workbench (NMDR)	National Institutes of Health (NIH)	Repository for metabolomics data and metadata	Public	~2,200 MS and NMR studies	~174,000 metabolite structures
GNPS/MassIVE	University of California San Diego	Natural product mass spectrometry data repository	Public	~490,000 mass spectrometry files, 1.2 billion tandem mass spectra	Not specified
Blood exposome database	UC Davis/Fiehn Lab	Chemicals detected in human blood specimens	Public	Not specified	41,474 achiral structures (65,957 PubChem CIDs)
Exposome-explorer	International Agency for Research on Cancer (IARC)	Biomarkers of exposure to environmental risk factors	Public	Not specified	908 dietary and pollutant biomarkers
METLIN exposome	Scripps Research Institute	Environmental toxicants, food contaminants, drugs identification	Public	Not specified	950,000 + unique small molecules
Human metabolome database (HMDB)	University of Alberta	Small molecule metabolites found in the human body	Public	Not specified	253,245 metabolites (3,444 detected and quantified)
UK biobank metabolomics	UK Biobank/Nightingale Health	Circulating metabolomic biomarkers in population cohort	Public (restricted access)	120,000 participants (expanding to 500,000)	249 metabolic measures
HELIX study	European Research Consortium	Environmental exposures during early life (pregnancy and childhood)	Public	31,472 mother–child pairs	Not specified
PeakForest	MetaboHUB/INRAE	Storage and annotation services for metabolic profiles	Public	Not specified	Not specified
T3DB (toxin database)	University of Alberta	Toxic compounds and their protein/DNA targets	Public	Not specified	3,700 toxic compounds linked to 2,086 protein/DNA targets
DrugBank	University of Alberta	Drug, drug-target and pharmaceutical information	Public	Not specified	11,891 drugs (4,563 FDA approved)
SMPDB	University of Alberta	Small molecule pathway database	Public	Not specified	55,700 metabolites (non-redundant)
FooDB	University of Alberta	Food component database	Public	Not specified	~24,000 food chemicals

Here we review some prominent ones and how they utilize (or could utilize) retention time and AI predictions:

### SIRIUS and CSI:FingerID

SIRIUS^12^ is a widely used framework for small molecule identification from MS and MS/MS data, focusing on molecular formula determination and fragmentation trees. CSI:FingerID (integrated with SIRIUS) then scores candidate structures by comparing predicted versus observed fragmentation patterns (53). Historically, SIRIUS/CSI did not use RT, focusing on spectral data. However, recent updates have added features to incorporate RT heuristics. The SIRIUS GUI can import retention times and even allows the user to apply a logP-based filter: it calculates an approximate XLogP for each candidate (using the Chemistry Development Kit) and provides a slider to filter candidates by logP range, which indirectly corresponds to an RT range. For example, if an unknown eluted at a very hydrophilic region, one might slide to only allow candidates with XLogP below a certain threshold (i.e., polar compounds). This is a rudimentary use of RT, but effective in pruning obvious mismatches. In future, we anticipate SIRIUS will integrate more advanced RT scoring—possibly by taking predicted RT (from an external model or a built-in one) and adding it to the overall score. A workflow developed in 2023 called COSMIC (Confidence of Small Molecule Identifications) already suggests combining *in silico* structure generation with retention filtering for higher confidence (52). COSMIC provides a breakthrough in high-confidence annotation of unknowns absent from spectral libraries by combining machine learning with probabilistic scoring. When applied to 17,400 metabolomics datasets, it recovered 1,715 novel structures with FDR control-enabling scale-compatible confidence filtering in exposomics. As SIRIUS development continues, users should watch for plugins or options related to RT. Even now, one best practice is: after obtaining a candidate list from SIRIUS/CSI, manually cross-check if the candidates’ predicted or known RTs align with the experimental RT, eliminating those that do not fit.

### MetFrag

MetFrag^13^ is an *in silico* fragmentation tool that scores candidates based on how well they explain the observed MS/MS peaks. Critically, MetFrag has been at the forefront of incorporating non-spectral information as additional scoring terms. The 2016 “MetFrag relaunched” version introduced the ability to use retention time in two ways: (1) Internal RT model—if the user provides a file of known compounds’ RTs in the same method, MetFrag will build a linear regression model between those compounds’ predicted logP and their RT. It then predicts a logP for each candidate and uses the regression to estimate an expected RT; candidates get penalized if their expected RT deviates from the observed RT of the unknown. (2) User-defined score—the user can separately compute any retention score (e.g., using an external QSRR model or simply an absolute difference from expected RT) and feed it into MetFrag as an additional column in the candidate list. MetFrag will then include that in the final score weighting. These features allow a flexible integration of RT. The impact is huge: as noted earlier, including retention data improved MetFrag’s top-rank identifications by many-fold. Using MetFrag with RT requires a bit more effort (one needs either reference data or an external predictor), but it is a highly recommended practice. For example, if analyzing a batch of plasma metabolites on an LC–MS, one can identify a subset of known metabolites first (using standards or library matches), fit a quick RT vs. logP curve, and then run MetFrag with that model to prioritize candidates for the unknowns. The result will favor chemically plausible candidates that fit the chromatography. MetFrag’s documentation and training materials provide guidance on how to format the RT training file or custom score input.

### HMDB (human metabolome database)

HMDB (54) is a rich database of human metabolites, including structures, concentrations, and in many cases experimental spectra. The Human Metabolome Database (HMDB 5.0) now catalogs over 217,000 annotated metabolites and more than 1.5 million derivatized entries, including predicted RT, MS/MS, and NMR data. These additions significantly enhance identification coverage in untargeted exposomics, especially for blood exposome compounds and microbial or food-derived metabolites identification. HMDB serves as a reference library—one can search by m/z or formula to find possible matches that are known human metabolites. While HMDB is not an identification software per se, it is invaluable for prioritizing biologically relevant candidates. Retention times in HMDB: HMDB does include some GC–MS and LC–MS spectral data for certain entries (e.g., from literature or experimental assays), and these often come with RT or RI information. For instance, HMDB’s MS spectra section may list a retention index for a metabolite’s GC spectrum, or an LC retention time if available. However, these are not standardized and only apply if the exact same method was used. HMDB 5.0 (2022 update) expanded content but still does not offer a unified retention index system. Instead of direct RT usage, HMDB’s role in our context is: provide a filtered search space (metabolites likely present in blood). If a candidate is in HMDB, it is more likely to be a real endogenous compound. Additionally, HMDB contains predicted properties (like logP, pKa) for many metabolites—those can be quickly accessed to sanity-check RT expectations. For example, if your unknown has an m/z matching glucose and another compound, and your chromatography is such that only a very polar compound would elute where it did, HMDB tells you glucose is highly polar (logP −3.24) whereas the other candidate is hydrophobic (logP +2). Such data, coupled with the observation, would favor glucose. In summary, HMDB is a database to cross-reference structures and their known data (though it does not perform the matching automatically, many analysis pipelines incorporate HMDB queries).

### PubChem

PubChem is the largest public chemical database, containing millions of compounds. Many identification workflows (including SIRIUS, MetFrag, and others) query PubChem for candidate structures by formula or mass. PubChem ensures we cast a wide net—the true identity could be a xenobiotic or unusual compound not in HMDB or other metabolic databases. However, PubChem provides almost no chromatographic data, as it is a general chemical repository. There are calculated properties (PubChem predicts logP, water solubility, etc., through its services), which could be used similarly to HMDB’s data for rough filtering. Some tools (like the PubChem search in MetFrag) can rank by the number of references, implying compounds commonly studied (e.g., drugs, natural products) rank higher. When using PubChem results, applying RT filtering is critical because the list can be enormous. This is where an automated RT prediction tool (like an QSRR model) is extremely useful: one can take all PubChem candidates and score them by RT fit to narrow down. So while PubChem itself does not aid directly with RT, it provides the candidate pool on which we apply our RT+AI filters.

### GNPS (global natural products social platform)

GNPS is primarily a platform for sharing and matching MS/MS spectra (especially for natural products and metabolites). It excels at *spectral networking*—grouping unknown spectra to known compounds or to each other based on similarity. GNPS’s library search can identify known compounds if an exact or similar spectrum is present in its extensive database. Retention time in GNPS: currently, GNPS spectral libraries do not systematically include retention indices; they focus on spectral data (though some contributors include a field for RT in their metadata). GNPS does incorporate optional retention time windows in some workflows—for example, when aligning features, one can set an RT tolerance to consider two features the same if their RTs are close. But in terms of identification, GNPS outputs a candidate match (with a spectral score) and often leaves it to the user to verify RT separately. In an unknown identification scenario, GNPS might tell you: “this spectrum best matches lysoPC(18:1) with score X.” It is then up to you to check if the RT is reasonable for a C_18_:1 lysophosphatidylcholine in your LC method. If you had a predictive RT model for lipids, you could confirm that. In fact, one study used a machine learning model to predict RT of lipids in an LC–HRMS lipidomics workflow and showed it helped confirm lipid annotations (62). So GNPS provides the spectral match, and an external RT model (or empirical expectation) provides an orthogonal check. We recommend incorporating RT filters when using GNPS outputs: for instance, if GNPS gives 5 candidate matches for an unknown, see if any have known or predicted RT close to your observation. Databases like LipidBlast^14^ or the Committee on Analytical Measurement (CITAC)^15^ Evaporative Light Scattering Detector (ELSD) retention index for lipids can be helpful reference points.

### Other tools/databases

There are many more resources in metabolomics. MassBank/MoNA (MassBank of North America)^16^ contains thousands of spectra often with retention information. A recent review compiled that MassBank (as of late 2023) had ~81,167 LC data records with RT and even 1,761 GC records with RI (5). These repositories could be mined to train better RT models or simply used to cross-check if an unknown’s RT matches a library entry for a given compound. PredRet (32) is a database specifically of experimental RTs across multiple systems, which was used to test RT projection methods. There are also vendor libraries (e.g., Agilent’s MassHunter Personal Compound Database and Library (PCDL)^17^ or Bruker’s HMDB library^18^) that include retention indices for GC or RT for LC under defined methods. These can directly support identification by matching your data to entries that have both spectral and RT matches. NIST’s Retention Index Database^19^ is another example of a specialized collection. For software, beyond MetFrag and SIRIUS, there is MS-DIAL^20^ and MS-FINDER^21^ which are free tools that also support retention indices for GC-EI data and have fields for LC retention (though they require user to input some reference values for LC). Retip^22^ (described earlier) is available as an R/Python tool to build RT prediction models with integrated databases—it is worth noting it comes pre-loaded with some retention libraries from Riken and UC Davis for certain conditions, which could be directly applicable if your method is similar. The Retip R package is a powerful open-source framework that integrates five different machine learning algorithms to predict RT with high accuracy across HILIC and RPLC. By incorporating Retip into MS-DIAL and MS-FINDER, Bonini et al. (55) achieved a 68% reduction in candidate annotations in a test dataset, demonstrating that coupling RT prediction with MS/MS scoring substantially improves metabolite identification confidence.

A comprehensive LC-Orbitrap screening workflow developed by Angeles et al. (56) highlights how stringent RT and MS2 filtering (5 ppm mass error, isotope presence, replicate consistency) can reduce false discovery rates in large-scale exposomics. Their detection of penilloic and penicilloic acid across six countries illustrates the utility of such workflows for global-scale chemical surveillance.

In conclusion, the ecosystem of tools and databases is increasingly supportive of multi-parameter identification. Best practice is to use a pipeline that combines complementary tools: for example, use SIRIUS/CSI or GNPS for MS/MS-based candidate generation, then use MetFrag or an in-house script to apply RT scoring to those candidates, utilizing databases like HMDB for biologically relevant filtering. Always cite the sources of your RT data or predictions when reporting an identification—this transparency helps build confidence in the result and allows others to reproduce the reasoning [e.g., “Compound X was identified as the likely structure because its predicted RT of 5.2 min matches the observed 5.0 min (within error), whereas other isomers had predicted RTs > 8 min”]. By leveraging the available software and DBs, analysts can vastly improve the throughput and reliability of unknown identification in untargeted metabolomics.

## Using known blood substances as internal calibrants

Calibrants dramatically improve RT transferability across labs. Zhang et al. (57) propose a post–projection calibration strategy that improves RT projection accuracy across chromatographic methods. Using 35 calibrants, their ReProjection model reduced projection errors below 3.2% and offers a generalizable route for integrating public RT resources into local annotation pipelines. However, these have to be added before measurements, so that they cannot help with existing measurements.

Human blood plasma and serum, the principal matrices of exposomics, offer a unique opportunity as they have a core set of metabolites that appear in virtually every sample (glucose, lactate, amino acids like alanine and glutamine, essential fatty acids, etc.). These known endogenous compounds can serve as convenient internal calibrants for retention time in each run. The concept is to take advantage of compounds already present in the sample matrix, whose identities can be confirmed (either via standards or strong database matches) and use them to adaptively model the RT behavior for that specific run or batch.

*Steps to utilize internal calibrants*:

1. *Identify a set of known metabolites in the sample*. These could be confirmed by running pure standards or by confident library matches. Priority should be given to compounds covering a range of RTs—e.g., an early-eluting polar metabolite (like citric acid), a mid-eluting one (like caffeine or tryptophan), and a late-eluting hydrophobic one (like cholesterol if doing broad lipidomic runs, or a long-chain fatty acid). The more points and the more spread-out they are, the better the calibration. In practice, even 5–10 compounds can be sufficient, as demonstrated by García et al. (4).
2. *Obtain their experimental RTs and theoretical RT predictions*. The theoretical predictions can come from a generic model (trained on a large dataset for that modality) or even from literature if the compound’s RT under identical conditions is known. Often, one might just use the model’s prediction (which could initially be off due to method differences, but that is fine). Now you have pairs of (predicted RT, observed RT) for each calibrant.
3. *Fit a calibration model*. This could be as simple as a linear regression (adjusting slope and intercept), or more complex (polynomial, or a warping function). Often a linear shift + scale is enough if the method differences are primarily gradient timing or flow rate differences (causing a nearly uniform shift/stretch of RT). For instance, if most compounds elute 20% earlier than predicted, a calibration might derive a factor to multiply predicted RTs by 0.8. Sometimes a second-order fit might capture slight curvature (if early and late “eluters” shift differently). In García et al.’s (4) meta-learning approach, they effectively learn a small Bayesian model for this mapping that also outputs uncertainty. The output is a function that can input any predicted RT and output a calibrated RT for the current run.
4. *Apply the calibrated model to all candidate predictions*. If you have a list of candidate structures (from formula matching), predict their RT with the base model, then adjust via the calibration function to get an estimated RT specific to your run. This yields what Figure 2 (above) calls the “projected database” of RTs.
5. *Compare unknown features to the calibrated predictions*. For each unknown feature with an observed RT, find which candidate structures have a projected RT near that value. One can calculate a difference or *z*-score (difference divided by the prediction error). Rank candidates by this score—smaller difference (within error) = better rank. If the difference is beyond a reasonable threshold, that candidate can be considered incompatible. Known substances in blood thus act as an *adaptive model*: if today the column is a bit slower, all predictions get shifted accordingly; if a different gradient is used, the model automatically compensates once it learns from the calibrants.

An example of this in practice: suppose an untargeted LC–MS of serum identifies metabolites like caffeine (RT 4.2 min observed) and leucine (RT 2.1 min). The global model predicted caffeine at 4.5 and leucine at 2.5 min for the standard gradient. The calibration fit might realize a factor of ~0.93 on RT (since both came out ~7–20% earlier). After calibration, the model predicts another compound, say hypotaurine, to elute at 1.0 min instead of 1.2 min it originally thought. If you indeed see a feature ~1.0 min that matches hypotaurine’s m/z, that bolsters the identification. Conversely, if a candidate was predicted (after calibration) to appear at 8 min but your unknown is at 3 min, you can drop that candidate.

One must be cautious that the calibrants themselves must be correctly identified (garbage in, garbage out). Thus, it is ideal to use high-confidence metabolites (possibly level 1 IDs confirmed with standards). Many labs include a mixture of authentic standards of common metabolites spiked into a representative matrix as part of their quality control—these can double as RT calibrants. Alternatively, natural ubiquitous metabolites suffice if you are confident in their annotation.

Another consideration is matrix effects: in complex blood extracts, very early or very late RT extremes might have fewer features. If your unknown falls outside the RT range covered by calibrants, extrapolation of the calibration model can be less reliable. To mitigate this, try to have calibrants near the boundaries of your chromatogram (e.g., a sugar that elutes at void volume, and a long-chain lipid that elutes near the end). If not, be more conservative in interpreting RT match for compounds beyond the calibrant range.

Using known blood metabolites as calibrants is essentially creating an *in situ* retention index system: instead of referencing an external standard mix, you reference inherent compounds. This has the benefit of no additional sample preparation and captures any matrix-induced shifts as well (since calibrants experience the same matrix). The Talanta study using acylcarnitines is a prime example—they leveraged metabolites naturally present to create a quantitative RT scale (endoRI) (43). We encourage metabolomics researchers to adopt similar strategies: for any dataset, list a few confidently identified compounds and use them to “lock” the RT scale of that dataset. This can be done *post hoc* too—for existing datasets, if you can retrospectively identify some features, you can recalibrate and re-search unknowns with improved accuracy.

## Advances in RT prediction models and their integration into workflows

Traditional QSRR models, such as linear regression or small nonlinear approaches using physicochemical descriptors (e.g., logP, polar surface area, hydrogen-bond donors/acceptors), offer the advantages of interpretability and require relatively few training compounds. However, their predictive accuracy tends to plateau, especially when applied across chemically diverse datasets. By contrast, deep learning and graph-based models (e.g., DNNs, RT-Transformer, GraphRT) exploit large-scale datasets such as SMRT or RepoRT to achieve far higher accuracy, often reducing prediction errors to mere seconds on RPLC gradients. These models can capture complex, nonlinear retention patterns and subtle structural effects (such as branching or isomerism), but function more as “black boxes” with limited mechanistic transparency.

An emerging middle ground is provided by transfer learning and meta-learning techniques, which retain the power of global models while adapting to local conditions with minimal additional data. For example, the Bayesian meta-learning approach described by García et al. (4) enabled recalibration of a global DNN predictor using only a handful of calibrants, consistently achieving cross-laboratory errors below 0.3 min. This represents a major practical benefit, as it reduces the need for each lab to retrain models from scratch while maintaining accuracy across diverse chromatographic systems.

The integration of calibration strategies also highlights trade-offs between RI-based approaches and model-based projection methods (Table 3). RI systems, such as Kovats indices for GC or chromatographic hydrophobicity index (CHI) for LC, provide absolute, portable scales that facilitate reproducibility and data sharing across laboratories. In contrast, model-projection strategies (e.g., z-score calibration using local calibrants) can more flexibly fine-tune global models to the specific conditions of a given experiment, often yielding higher within-study accuracy. Endogenous calibrants (endoRI), such as acylcarnitines in plasma, further extend this concept by leveraging compounds inherently present in biological samples to anchor RT scales.

**Table 3:** Use of retention time (RT/RI) in major annotation tools and workflows.

Tool/workflow	RT/RI input capability	Use of RT in scoring/ranking	Calibration or RT alignment options	Outputs/integration
MetFrag (30)	Accepts observed RT; can incorporate predicted RTs (from QSRR/ML).	RT deviation penalizes candidates; requires external predicted RT source.	No internal calibration; external RT model calibration recommended.	Ranked list of candidates with composite score (can include RT).
SIRIUS/CSI:FingerID (SIRIUS manual)	No direct RT input; LogP slider acts as a proxy filter.	N/A (filter only); user can exclude candidates inconsistent with expected RT.	None.	Ranked formulas/structures by MS/MS score only.
KGMN Zhou et al. (61)	Uses chromatographic co-elution patterns between known and unknown features; does not use absolute RT values.	RT contributes indirectly (binary co-elution link); no numerical RT scoring.	Not applicable (relative within-sample).	Annotation network linking unknowns to known metabolites; confidence scores, no explicit RT score.
COSMIC (52)	RT not used in scoring; users can filter externally.	Confidence score based on MS/MS fragmentation and metadata; no RT term.	None.	Candidate list with ML-based confidence score; RT verification possible post hoc.

MetFrag is currently the most flexible in incorporating RT explicitly, provided users supply predicted RTs or calibrants. SIRIUS/CSI:FingerID offers an indirect proxy via logP filtering but does not yet integrate RT in scoring. KGMN leverages relative RT through co-elution rather than absolute values. COSMIC focuses on large-scale confidence scoring without RT, though external RT filtering can complement its workflow.

Taken together, these comparisons underscore that no single approach is universally superior. Instead, QSRR and AI-based methods, index systems and projection approaches, offer complementary strengths: interpretability versus accuracy, reproducibility versus flexibility. Explicitly articulating these trade-offs helps ensure that researchers adopt RT-aware workflows that are both scientifically robust and practically feasible across different experimental settings.

## Best practices and recommendations

Bringing together the insights above, here are best-practice recommendations for improving unknown metabolite identification by integrating retention time, QSAR models, and AI predictions:

- *Incorporate RT from the start*: Always record accurate retention times for all features during LC–MS/GC–MS data acquisition (ensure your data processing exports RT for each peak). Treat RT as a standard part of the feature annotation (just like m/z and MS/MS spectrum).
- *Use multi-modal evidence in identification*: Do not rely on m/z or spectral match alone. Use retention time as a critical filter or scoring factor. If a library hit has a vastly different RT (or RI) than your unknown, consider it suspect even if spectra match. Conversely, if an unknown’s RT matches a predicted or literature RT for a candidate, give that candidate a higher priority.
- *Build or apply RT prediction models appropriate to your chromatography*: If working in RPLC, you can leverage published models (e.g., those trained on SMRT). Tools like Retip allow you to train a custom model using ~300 known compounds—consider doing this if you have a rich in-house library. For HILIC or other less common systems, try transfer learning from a base model or gather a small training set of standards. Always test the model’s accuracy on a handful of knowns to gauge its error.
- *Calibrate retention times for each run/batch*: Implement a retention index or calibration scheme. For LC, you can simply inject a standard mix (e.g., a mixture of amino acids or a commercial RT calibration mix) or rely on endogenous calibrants as discussed. Perform calibration early in your data analysis. For GC, always calculate retention indices for unknowns using alkane standards (if your method allows)—most GC libraries expect RI for matching.
- *Leverage software capabilities*: Use MetFrag’s retention time scoring option if doing *in silico* fragmentation ranking. In SIRIUS, use the logP filter slider to remove implausible candidates (and stay tuned for more RT integration in future versions). In MS-DIAL or other pipelines, make use of any retention time alignment and annotation features—for instance, MS-DIAL can match features to a database with RT constraints if you provide one.
- *Utilize databases and literature*: Before assigning an ID, check resources like HMDB or publications for reported retention times of that metabolite under similar conditions. For GC, compare your measured RI to known RI values (NIST webbook or literature)—a match within ~ ± 10 index units strongly supports the ID. For LC, if a metabolite was previously identified in a similar method, use that as supporting evidence (keeping in mind method differences).
- *Combine AI predictions with expert knowledge*: AI models are powerful, but still benefit from chemical intuition. If a model predicts an RT that seems off given known behavior (e.g., it predicts a very polar molecule to elute extremely late on C_18_), double-check and consider alternative models or descriptor checks. Use predicted properties (logP, pKa) to sanity-check: e.g., if a candidate is extremely hydrophobic but your unknown eluted early in a polar fraction, that is a red flag.
- *Internal standard usage*: In prospective studies, include a set of internal standards spanning the polarity range. Isotopically labeled versions of metabolites can be ideal as they co-elute with natives. These will ensure precise RT references and also help monitor any chromatographic drifts during the run.
- *Continuous model validation*: As you confidently identify more compounds in your dataset, iteratively feed those back to refine your RT model. This adaptive approach (akin to active learning) can improve predictions for the remaining unknowns. Many of the mentioned tools allow dynamic addition of calibrants or re-training.
- *Document and report the RT evidence*: When publishing or reporting identifications, note the retention time and how it was used. For example: “*Compound X was putatively identified as Y; supporting evidence includes an observed RT of 5.3 min, which closely matches the predicted RT of 5.1 min (error ~4%) for Y on our C_18_ method. Other isomers had predicted RTs of >8 min, making them unlikely.*” This not only justifies the ID but also contributes to collective knowledge of RT data.
- *Stay updated with new models and tools*: The field is evolving – new deep learning models (like retention time transformers, graph neural nets) are being published. New databases (like expanded MassBank (59), PredRet (32) updates, etc.) are coming online with more retention data. Keep an eye on these developments, as they can be quickly applied to improve your analyses. For instance, if a new model drastically improves HILIC RT prediction, adopting it could resolve some ambiguous IDs that were previously uncertain.

To promote reproducibility and harmonization of RT-informed metabolomics workflows, we recommend that authors include the items listed in Box 1 when reporting retention time calibration.

Box 1 **Retention time calibration checklist.**

1. Calibration compounds and range

- List the internal or external calibrant compounds used, including their identities and concentrations (or source, if endogenous).
- Report the retention time span they cover, ensuring early- and late-eluting compounds are included.

2. Chromatographic method details

- Specify stationary phase (column chemistry, dimensions), mobile phase gradient program, flow rate, column temperature, and buffer composition/pH.
- These parameters are critical for contextualizing retention times and enabling reproducibility.

3. Calibration function/model

- Describe the mathematical function used to relate predicted and observed RT (e.g., linear regression, polynomial fit, retention index scale).
- Note if a *z*-score or probabilistic correction was applied.

4. Calibration performance

- Report residuals or error statistics for calibrants (e.g., RMSE, median absolute error, R2).
- Provide a statement such as: “Calibration achieved residuals <0.2 min for 95% of calibrants.”

5. Reference scale or index

- Indicate whether a standardized retention index (e.g., Kovats RI, CHI) or endogenous retention index (endoRI) system was used.
- State the reference compounds (e.g., n-alkanes, acylcarnitines).

6. Quality control information

- Document how calibration was checked over time (e.g., across batches, runs, or instruments).
- If calibration stability was monitored, report any drift or corrections applied.

By following these practices, researchers can achieve more accurate and confident identifications in untargeted metabolomics and exposomics (see Box 2). The combination of high-resolution MS data with AI-driven RT prediction and proper calibration constitutes a powerful approach to tackle unknown features. What used to be a bottleneck (having tens of thousands of “unknown unknowns” in a metabolomic profile) can gradually be alleviated by systematically narrowing possibilities and pinpointing structures that make sense both in mass *and* in chromatographic behavior.

Box 2 **Getting started tomorrow: integrating RT into your metabolite ID workflow.**

1. *Record all retention times*: ensure your LC–MS or GC–MS data processing exports accurate RTs (or RIs) for every detected feature. Treat RT as a standard piece of metadata for each peak, just like m/z or MS/MS spectrum.
2. *Calibrants at hand*: select a set of reference compounds (endogenous metabolites or spiked standards) that span the chromatographic range – from early to late eluters. Run these in your system to serve as RT calibrants.
3. *Pick a prediction tool*: choose a QSRR/RT prediction model suitable for your needs. You can use open tools (e.g., Retip in R, or published deep learning models) and/or train a simple model using ~20–50 known compounds from your lab’s library.
4. *Calibrate and predict*: apply the model to predict RTs for candidate structures. Use your reference compounds to calibrate or align predictions to your instrument (simple linear fit or retention index). Then for each unknown, compare its observed RT to predicted RTs—filter out candidates that fall outside an acceptable window (e.g., > ± 5% deviation).
5. *Verify and report*: for top candidate IDs, cross-check against literature or databases for matching RT (under similar conditions). Document how RT was used to support identifications in your report or supplementary info (include predicted vs. observed RT, calibration details, etc.). This transparency will strengthen confidence in your results.

## Limits and caveats

While RT prediction has advanced substantially, several limitations and edge cases remain important for practical implementation. Awareness of these applicability domain (AD) constraints can help researchers avoid misinterpretation and overconfidence in predictions.

### Non-retained compounds in RPLC

Highly polar or ionic compounds may be essentially unretained in reversed-phase LC, eluting near the void volume. Such early-eluting features fall outside the useful range of most RPLC QSRR models, and predictions in this regime are often unreliable. Aalizadeh et al. (31) addressed this issue by developing a classifier to flag non-retention on C18 columns, illustrating that a dedicated check for non-retention is feasible. In practice, we recommend that analysts first assess whether a compound is likely to be unretained (e.g., predicted to elute at <0.5 min or with extremely high polarity) before applying an RPLC-based RT prediction.

### Ion-pair and ion-exchange chromatography idiosyncrasies

Specialized chromatographic modes such as ion-pairing LC or ion-exchange LC rely on unique ionic interactions that general RPLC models cannot capture. Even subtle changes in conditions (e.g., mobile phase pH, buffer composition, salt concentration, counter-ion identity) can dramatically shift retention times, narrowing the applicability domain for predictive models. Consequently, if an unknown was analyzed under ion-pair or ion-exchange conditions, a standard RPLC model should not be applied blindly. Instead, a mode-specific model or at minimum a locally calibrated approach is required. Users should verify that the model’s training data include similar ionic conditions, or consider re-training with appropriate calibrants before relying on predictions.

### Chemical-space distance versus error growth

RT prediction models, like other QSAR/QSRR frameworks, are most reliable within the chemical space they were trained on. Prediction error tends to grow as a compound’s structure diverges from the training set, for example in the case of novel scaffolds or extreme physicochemical properties. Users can apply simple AD checks, such as examining whether a compound’s descriptors fall within the training range, or by calculating leverage values in PCA space. Several modern tools (e.g., Retip, QSRR Automator) provide automated AD warnings or prediction confidence intervals; if an unknown has a very high uncertainty or lies outside the AD, its RT prediction should be interpreted cautiously. These checks help avoid misidentification due to model overreach.

### Extrapolation beyond calibrant range

Calibration strategies should not be extrapolated beyond the range covered by calibrants. For example, if calibrants span 1–10 min but a compound elutes at 12 min, the calibrated model is operating in extrapolation mode, where errors may be large and unpredictable. To minimize this risk, calibrants should bracket the entire chromatographic run, ideally including an early-eluting polar compound (near the void) and a late-eluting hydrophobic compound (near column wash). If such coverage is not achievable, identifications outside the calibrant range should be treated with greater caution. The inclusion of a calibrant panel spanning the RT range provides a practical safeguard against over-extrapolation.

Taken together, these caveats emphasize that RT prediction, while powerful, is not universally reliable. Proper consideration of chromatographic modality, retention regime, chemical space, and calibrant coverage ensures more robust application and prevents over interpretation of results.

## Conclusion

Untargeted metabolomics is entering an era where integrated data analysis—combining m/z, MS/MS, retention time, and even ion mobility—is the norm for rigorous metabolite identification. Chromatographic retention time, once considered a secondary or even nuisance parameter, is now recognized as an independent structural signature that can greatly aid identification when used intelligently. By developing RT prediction models (QSRR models and modern ML predictors) and calibrating them to specific experimental conditions, we can exploit RT to rank candidates and reduce false positives. This is a transformative improvement: studies show major boosts in correct identification rates when RT information is included in annotation workflows. The approach spans all chromatographic modalities—from reversed-phase and HILIC in LC to gas chromatography—each benefiting from tailored models and calibration techniques. AI and deep learning methods, empowered by large datasets, are key enablers, delivering accurate predictions and even the flexibility to transfer those predictions across different chromatographic setups.

Crucially, the tools to implement this strategy are increasingly at our disposal: algorithms like RT-Transformer for retention prediction, platforms like SIRIUS and MetFrag that incorporate multi-criteria scoring, and databases like HMDB and GNPS providing reference points. As we have outlined, using known blood metabolites as internal RT calibrants is a practical way to adapt these innovations to real-world samples without extensive extra work—your sample inherently contains a roadmap for RT alignment if you know where to look.

When incorporated into candidate ranking algorithms, RT prediction significantly improves annotation confidence. In landmark studies, correct metabolite IDs were recovered among top-ranked candidates in 68–86% of cases when RT predictions were included—compared to far lower performance using mass-based scores alone. This leap in performance is especially impactful for metabolites with few or no spectral matches in existing databases, which is often the case in exposome research.

To fully leverage RT as an identifier, however, three key developments are needed: (1) *Reliable RT Prediction Models Across Modalities*: Deep learning architectures, such as RT-Transformer and graph neural networks, have achieved mean absolute errors of <30 s for RP-LC and promising transferability to HILIC and GC systems. These models allow structure-based RT prediction even for compounds lacking experimental RT entries, including emerging contaminants. (2) *Run-Specific Calibration for RT Transferability*: Because RTs shift between instruments and over time, the development of robust calibration strategies using endogenous metabolites (e.g., acylcarnitines, amino acids) as internal calibrants is essential. Methods such as Bayesian meta-learning and endogenous retention index (endoRI) scaling correct systematic bias and enable comparison across datasets. (3) *Integration with Candidate Scoring Frameworks*: Platforms like MetFrag and SIRIUS now support RT filtering or scoring, enabling users to weight candidate structures by predicted RT proximity. This approach is especially effective when MS/MS spectra are absent, and it harmonizes with suspect screening workflows increasingly used in environmental exposomics.

In conclusion, we recommend that all untargeted metabolomics and exposomics studies adopt a mindset of integrative identification—leveraging retention time alongside spectral data and leveraging AI-based QSAR models for prediction. By doing so, the community will accelerate the identification of “unknown” features, improve the consistency of metabolite annotation across labs, and ultimately extract more biological insight from metabolomics data. With best practices in place, retention time will no longer be an underused feature, but rather an indispensable element of the metabolomics identification toolkit, supported by QSAR, AI, and sound analytical calibration. This holistic approach positions us to tackle the long tail of unknown metabolites with greater confidence and accuracy than ever before.

## Footnotes

^1^ https://web.ornl.gov/sci/techresources/Human_Genome/publicat/BattelleReport2011.pdf

^2^ https://eirene.eu

^3^ https://humanexposome.net

^4^ https://www.nexus-exposomics.org

^5^ https://exposomemoonshot.org

^6^ https://exposomemoonshot.org/washington-d-c-declaration-on-the-human-exposome/

^7^ https://www.mqacc.org

^8^ https://github.com/ipb-halle/MetFragTraining

^9^ https://fiehnlab.ucdavis.edu/projects/fiehnlib

^10^ https://www.hmdb.ca

^11^ https://github.com/AspirinCode/sirius

^12^ https://github.com/sirius-ms/sirius

^13^ https://ipb-halle.github.io/MetFrag/

^14^ https://fiehnlab.ucdavis.edu/projects/lipidblast

^15^ https://www.eurachem.org/images/stories/Guides/pdf/QUAM2012_P1.pdf

^16^ https://mona.fiehnlab.ucdavis.edu/

^17^ https://www.agilent.com/cs/library/usermanuals/public/G3336-90030_PCDLManager_QuickStart.pdf

^18^ https://store.bruker.com/products/bruker-hmdb-metabolite-library-2-0

^19^ https://chemdata.nist.gov/dokuwiki/doku.php

^20^ https://systemsomicslab.github.io/compms/msdial/main.html

^21^ https://systemsomicslab.github.io/compms/msfinder/main.html

^22^ https://www.retip.app
